# Clear cell sarcoma of the kidney with inferior vena cava thrombus: a case report

**DOI:** 10.1186/s13256-022-03489-2

**Published:** 2022-08-01

**Authors:** Leen Jamel Doya, Khaled Alyousef, Majdy Oukan, Ali Razzok, Basel Shek Alshabab, Tarek AlEid, Rabab Saloum, Hassan Nasser

**Affiliations:** 1 Department of Pediatrics, Faculty of Medicine, Tishreen University, Lattakia, Syria; 2 Department of Urology, Faculty of Medicine, Tishreen University, Lattakia, Syria; 3 Department of Pathology, Faculty of Medicine, Tishreen University, Lattakia, Syria

**Keywords:** Clear cell sarcoma of the kidney, Hematuria, Vascular tumor thrombus

## Abstract

**Background:**

Clear cell sarcoma of the kidney is an uncommon pediatric renal malignant neoplasm that is typically characterized in 2–3-year-olds by aggressive behavior and late relapses. Our literature review revealed fewer than ten previously reported cases of CCSK with inferior vena cava thrombus, with only five in the pediatric age group.

**Case presentation:**

We report the case of a 14-year-old Syrian girl who complained of mild pain in the left lumbar region pain with hematuria. On physical examination, a mass was palpated in the left flank. Abdominal ultrasonography revealed a left renal mass (7 × 5 × 2 cm^3^), associated with dilatation of the left renal vein. Contrast abdominal computed tomography showed a mass measuring 7 × 5 × 3 cm^3^ with the presence of thrombus extending into the inferior cavity down to the right atrium that was initially diagnosed as Wilms’ tumor. Radical right nephrectomy with excision of the thrombus was undertaken. Histological immunostaining revealed a diagnosis of the tumor as clear cell sarcoma with vascular tumor thrombus extending to the inferior vena cava.

**Conclusion:**

Clear cell sarcoma and Wilms’ tumor are similar in terms of typical age of appearance, clinical features, and histopathology, but with different methods of treatment and prognosis. The differential diagnosis of such masses is thus very important. We present the case of a patient with clear cell sarcoma with unusual age, with complete removal of the thromboses in the inferior vena cava and the right atrium.

## Introduction

Clear cell sarcoma of the kidney (CCSK) is an uncommon pediatric renal malignant neoplasm (representing approximately 2.8% of all primary renal tumors in children) [1]. CCSK is most common in children between 2 and 3 years old, with a slight predominance in males (male-to-female ratio of approximately 2:1) [2]. The most common symptoms of CCSK patients include abdominal pain, distension or mass, nausea, vomiting, weight loss, low-grade fever, hematuria, and anemia. It is known to show aggressive behavior and late relapses [3].

## Case presentation

A 14-year-old Syrian female was admitted to the Urology Department of Tishreen University Hospital with 2-month history of mild pain in the left lumbar region pain with hematuria and weight loss of over 10 kg in that interval. She was treated with antibiotics without improvement. There was no previous medical or familial history.

On physical examination, her temperature was 38.3 °C, oxygen saturation 98%, and arterial blood pressure was 11/8 (systolic pressure/diastolic pressure). She was stable with normal physical examination except for pallor and left lumbar region mass.

On laboratory analysis, erythrocyte sedimentation rate (ESR) and lactate dehydrogenase (LDH) were elevated. Complete blood count (CBC), C-reactive protein (CRP), renal and liver function, and glucose were normal.

Urinalysis showed 50–60 red blood cells (Table 1).

**Table 1:** Laboratory data of the case

Test	Result	Normal range	Test	Result	Normal range
WBC (10^3^/μL)	5.0	6.2–17	AST (U/L)	36	5–40
Neutrophils (%)	56	40–60	ESR (1 hour) (mm/hour)	20	0–10
Lymphocyte (%)	36	20–40	Glucose (mg/dL)	90	70–100
Hb (g/dL)	11.5	11–13	LDH (U/L)	852	60–170
MCV (fL)	66	70–85	Urea (mg/dL)	33	15–36
PLT (10^3^/μL)	322	150–450	Creatinine (mmol/L)	0.2	0.5–1.3
CRP (mg/dL)	3	< 5	K (mmol/L)	3.9	3–4.5
ALT (U/L)	39	7–55	Na (mmol/L)	133	135–145

*WBC* white blood cells, *HB* hemoglobin, *MCV* mean corpuscular volume, *RDW* red cell distribution width, *PLT* platelets, *CRP* C-reactive protein, *ALT* alanine aminotransferase, *AST* aspartate aminotransferase, *ESR* erythrocyte sedimentation rate, *LDH* lactate dehydrogenase

**Fig. 1:**
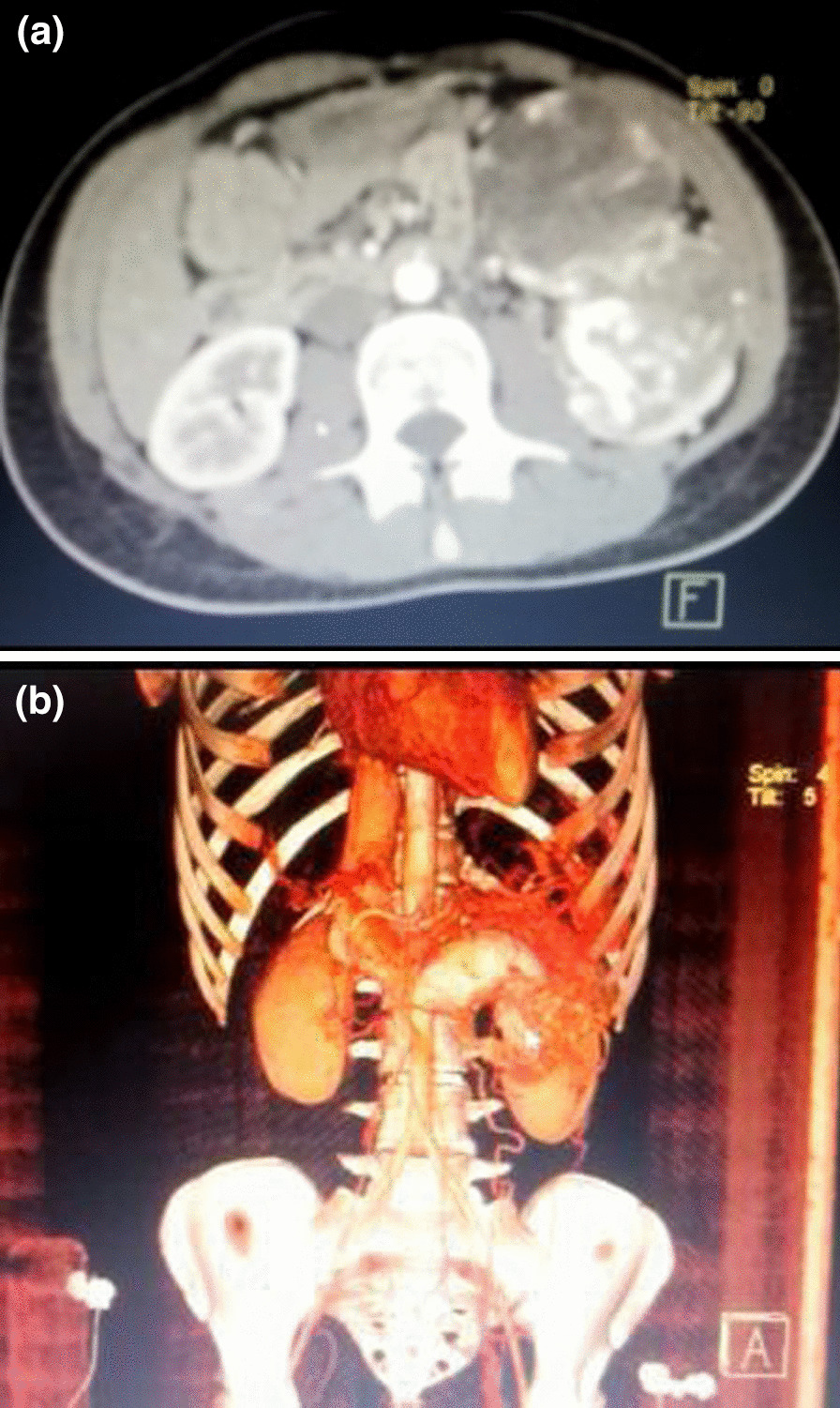
**a** Computed tomography (CT) scan of the abdomen showing a 7 × 5 × 3 cm^3^ mass arising from the anterior part of the left kidney with tumor thrombus in the inferior vena cava extending to the right atrium without metastases. **b** CT scan of the abdomen with reconstruction

Ultrasonography showed deformation of the left kidney measuring 7 × 5 × 2 cm^3^ with dilation of the left renal vein. Intravenous contrast computed tomography (CT) scan of the abdomen showed a mass with size of 7 × 5 × 3 cm^3^ arising from the anterior part of the left kidney with tumor thrombus in the inferior vena cava extending to the right atrium without metastases (Fig. 1a, b). Doppler echocardiography did not show a tumor thrombus into the right atrium.

**Fig. 2:**
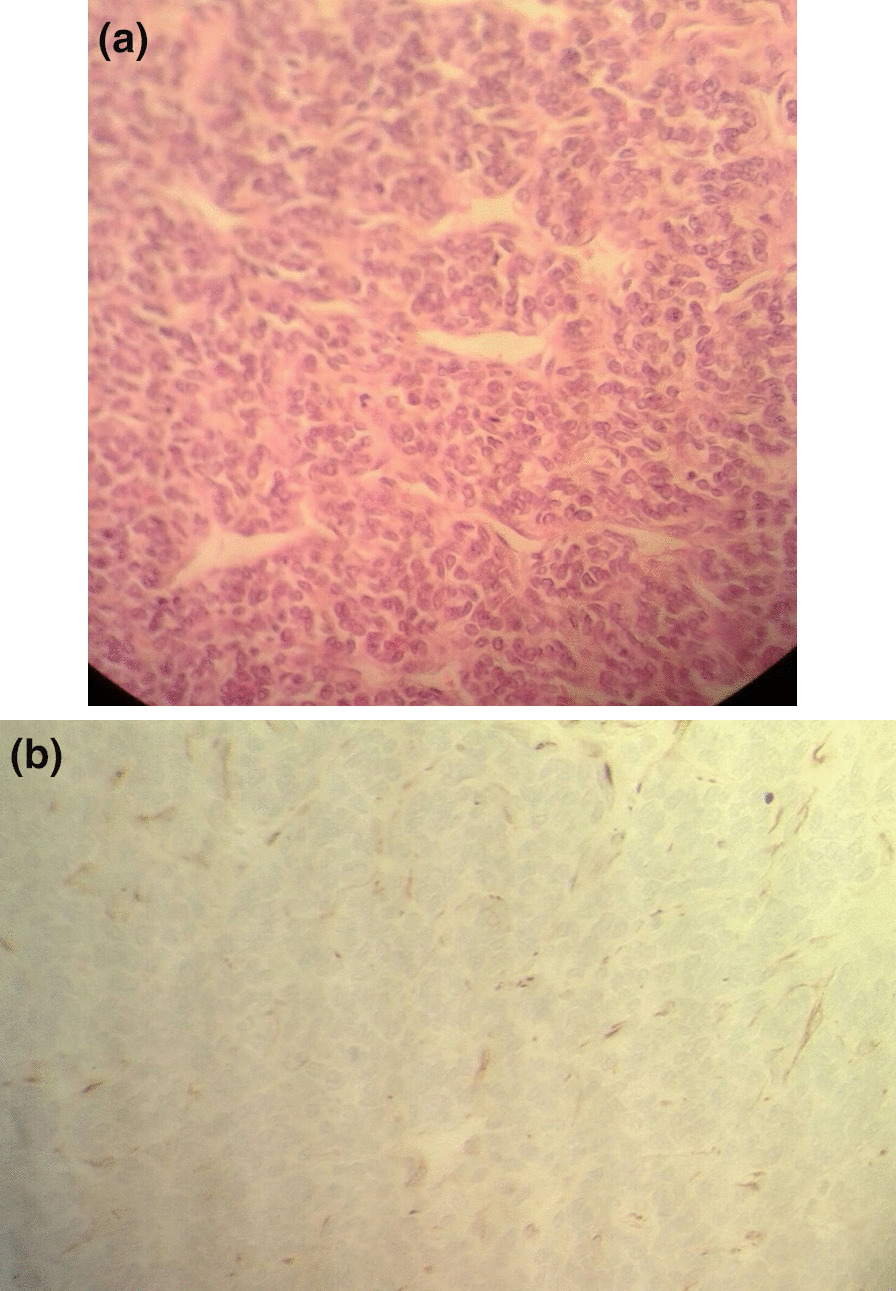
**a** Immunohistopathology of resected renal mass revealed vimentin positivity. **b** Immunohistopathology of the resected renal mass revealed negative cytokeratin

The operation proceeded by laparotomy midline abdominal incision. The exploration of the abdominal cavity showed a normal right kidney with normal liver. In the first stage, the right renal pedicle, inferior vena cava, hepatic vein, and lumbar veins were isolated. Then, left renal artery and vein were ligated, and left nephrectomy with lymphadenectomy of regional and paraaortic was carried out. Vessel loops were placed at the iliac branching, the hepatic vein, the right renal artery and vein, and over the incision on the inferior vena cava vascular to remove the tumor thrombus. Venotomy on inferior vena cava vascular was performed to excision the tumor thrombus by inserting a clump of blood suction device (Sil Silver) with monitoring of the patient’s vital signs (pulse, blood pressure, and oxygen saturation). The patient was systemically heparinized in the intensive care unit. The postoperative course was uneventful; after 72 hours, she was referred to the urology department. Seven days later, she was discharged from the hospital in good condition. The histopathology of the resected renal mass revealed a tumor cell with clear cytoplasm, indistinct nucleoli, and abundant extracellular matrix. There were cystic and focal areas of necrosis (Fig. 2). The regional and para-aortic lymph nodes did not show any involvement. Immunostaining was vimentin-positive but negative for CD 99, CD34, pan CK, desmin, CK7, cytokeratin, CD 10, WT 1, EMA, NSE, and chromogranin A. the final diagnosis of CCSK with inferior vena cava was made. At 15 days postoperatively, the patient was treated with chemotherapy containing vincristine, doxorubicin, cyclophosphamide, and etoposide. We followed the patient for 12 months after surgery; she remains stable without any recurrence or metastasis.

## Discussion

The first use of the term CCSK was in 1970 by Beckwith and Palmer. In the same year, Kidd first reported it as a separate clinicopathological entity [4]. CCSK most commonly metastasizes to bone (13%), lymph nodes (59%), lung (10%), and liver (9%). It can rarely invade the inferior vena cava with extension into the right atrium [1]. Our literature review revealed fewer than ten previously reported cases of CCSK with inferior vena cava thrombus, with only five in the pediatric age group (Table 2).

**Table 2:** Literature cases of pediatric CCSK with inferior vena cava thrombus

Author/date	Patient	Manifestation	Diagnosis	Treatment
Nazl (2014)	An 18-year-old female with CCSK in the right kidney, with a thrombus extending to the inferior vena cava	Right-sided abdominal mass	Radiological features (transthoracic echocardiography, CT) with histopathological study	Surgical intervention, chemoradiotherapy
Hiradfar (2012)	A 6-year-old girl with CCSK of right kidney with atriocaval thrombus	Common cold symptoms, on physical examination flank mass in the right side of the abdomen	Radiological features with ultrasound-guided fine-needle biopsy, histopathological study	Chemotherapy with surgical intervention
Sugandhi (2011)	A 3-year-old boy with CCSK of the right kidney with cavoatrial thrombus	Gradually progressive right-sided abdominal mass of 7-month duration	Echocardiography, ultrasound-guided needle biopsy, histopathological study	Neoadjuvant chemotherapy, radical nephrectomy, lymphadenectomy, intravascular tumor, resection, postoperative chemotherapy
Zigman (2006)	A 23-month-old girl with CCSK of right kidney with cavoatrial tumor thrombus	Increased abdominal girth over the previous month, abdominal mass, microscopic hematuria	Echocardiography, ultrasound-guided needle biopsy, histopathological study	Chemotherapy with surgical intervention
Ohtake (1995)	A 6-year-old boy with CCSK of the right kidney extending into the inferior vena cava	Right flank mass, macrohematuria	Radiological features with histopathological study	Radical nephrectomy, lymphadenectomy, intravascular tumor, resection, postoperative chemotherapy

All of the patients identified by the literature review had right masses, while in this case, the patient had a left kidney mass.

No laboratory studies can confirm the diagnosis of CCSK. Laboratory investigations including complete blood count, inflammatory markers such as CRP, and erythrocyte sedimentation rate are nonspecific [1]. Radiological assessment of CCSK shows no features that distinguish it from other renal tumors [4].

Doppler ultrasonography, contrast-enhanced computed tomography, and MR angiography are the techniques that can detect intracaval and intraarterial thrombus. Besides, an echocardiogram may provide additional information regarding the extent of the interatrial thrombus. The major diagnostic method that provides the final diagnosis remains histopathologic examination [5]. Regarding the pathological diagnosis of CCSK, it is difficult to distinguish CCSK from blastomal and stromal Wilms’ tumor (WT). The typical histopathological characteristics of CCSK are cystic formation, necrotic foci, and large size with a mucinous combination. It is composed of nests of cells with scant cytoplasm and a high nuclear-to-cytoplasmic ratio. The nuclei are mitotic structures with fine chromatic patterns [6]. Immunohistochemistry can help to distinguish CCSK from other renal tumors. It shows nonspecific positivity for vimentin but is negative for Mic-2, WT-1, desmin, cytokeratin, epithelial membrane antigen, and S100 [1].

In a review of multiple, large case series, Cooskens *et al.* reported the staging of CCSK and its prevalence as presented in Table 3 [1]. According to this staging, our patient had stage III disease.

**Table 3:** Literature cases of pediatric CCSK with inferior vena cava thrombus

Stage of CCSK	Description	Treatment
Stage I (27%)	The tumor is limited to the kidney, being less than 7 cm with an intact capsule and no evidence of rupture The vessels of the renal sinus are not involved	Complete resection
Stage II (33%)	The tumor extends beyond the kidney, measured as more than 7 cm with regional extension of the tumor; blood vessels outside the renal parenchyma (including those of the renal sinus) may contain tumor	Complete resection
Stage III (34%)	The tumor is found within the kidney parenchyma or blood vessels, and in surrounding lymph nodes and fatty tissue. Gross or microscopic evidence of the tumor is present after resection	Incomplete resection
Stage IV (6%)	Tumor spread beyond the kidney with hematogenous metastases (lung, liver, bone, brain) or lymph node metastases extending beyond of the abdominopelvic region	Incomplete resection
Stage V	Bilateral renal involvement discovered at diagnosis	Resection not possible

Treatment of CCSK remains controversial, and the optimal treatment is unknown. A combination of chemotherapy regimens (cyclophosphamide, doxorubicin, actinomycin D, and vincristine), and radical nephrectomy with or without radiation is used [7].

According to our literature review, the therapeutic strategy for intravascular thrombosis in the cases of pediatric tumors is based on preoperative chemotherapy to achieve intravascular tumor regression with delayed resection of the intravascular thrombosis during a laparotomy [8]. Pediatric CCSK cases show poor response to chemotherapy, and complete surgical excision of the primary tumor with its thrombus extension along the inferior vena cava is the appropriate treatment option [9].

The present case report describes a patient with CCSK with vena cava thrombus. Initially, surgical treatment followed by chemotherapy was believed to be the treatment of choice.

## Data Availability

All data generated or analyzed during this study are included in this published article.
